# Differentiation of human‐induced pluripotent stem cell under flow conditions to mature hepatocytes for liver tissue engineering

**DOI:** 10.1002/term.2659

**Published:** 2018-04-06

**Authors:** Viktoriia Starokozhko, Mette Hemmingsen, Layla Larsen, Soumyaranjan Mohanty, Marjolijn Merema, Rodrigo C. Pimentel, Anders Wolff, Jenny Emnéus, Anders Aspegren, Geny Groothuis, Martin Dufva

**Affiliations:** ^1^ Department of Micro‐ and Nanotechnology Technical University of Denmark Denmark; ^2^ Groningen Research Institute for Pharmacy University of Groningen Groningen The Netherlands; ^3^ Cellartis, Takara Bio Europe AB Gothenburg Sweden

**Keywords:** bioartificial liver, hepatic differentiation, stem cells

## Abstract

Hepatic differentiation of human‐induced pluripotent stem cells (hiPSCs) under flow conditions in a 3D scaffold is expected to be a major step forward for construction of bioartificial livers. The aims of this study were to induce hepatic differentiation of hiPSCs under perfusion conditions and to perform functional comparisons with fresh human precision‐cut liver slices (hPCLS), an excellent benchmark for the human liver in vivo. The majority of the mRNA expression of CYP isoenzymes and transporters and the tested CYP activities, Phase II metabolism, and albumin, urea, and bile acid synthesis in the hiPSC‐derived cells reached values that overlap those of hPCLS, which indicates a higher degree of hepatic differentiation than observed until now. Differentiation under flow compared with static conditions had a strong inducing effect on Phase II metabolism and suppressed AFP expression but resulted in slightly lower activity of some of the Phase I metabolism enzymes. Gene expression data indicate that hiPSCs differentiated into both hepatic and biliary directions. In conclusion, the hiPSC differentiated under flow conditions towards hepatocytes express a wide spectrum of liver functions at levels comparable with hPCLS indicating excellent future perspectives for the development of a bioartificial liver system for toxicity testing or as liver support device for patients.

## 1 INTRODUCTION

Even though fully matured primary human hepatocytes (PHH) exhibit all the specific liver functions, their limited availability and loss of liver‐specific functions during culturing in vitro are still the major limitations for their application in a bioartificial liver (BAL; Mizumoto et al., 2012; Ordovás, Park, & Verfaillie, 2013). Therefore, porcine primary hepatocytes and carcinoma cell lines (HepG2, HepG2/C3A, and HepaRG) have been widely employed in liver engineering (Nibourg, Chamuleau, van Gulik, & Hoekstra, 2012; Palakkan, Hay, Anil Kumar, Kumary, & Ross, 2013; Schwartz, Fleming, Khetani, & Bhatia, 2014). The drawbacks of these cells are however the risk of zoonotic diseases, immunological responses, tumour formation, or poor liver‐specific functions compared with PHH (Nibourg et al., 2012; Palakkan et al., 2013).

Stem cells, especially human‐induced pluripotent stem cells (hiPSCs), have therefore received great attention during the past years for liver tissue engineering (Nibourg et al., 2012; Ordovás et al., 2013). hiPSCs represent a potentially unlimited cell source for a large‐scale production of hepatocytes required for BAL development. Furthermore, the use of the patients' own hiPSCs may allow for personalized treatment and thereby avoiding immunological reactions. Although hiPSC‐derived hepatocyte‐like cells have been shown to have certain liver‐specific phenotypic characteristics and exhibit many of the liver‐specific functions (Chen et al., 2012; Gieseck et al., 2014; Si‐Tayeb et al., 2010; Song et al., 2009), most of these functions are expressed at levels several magnitudes lower than in fresh liver tissue or freshly isolated human hepatocytes (Ulvestad et al., 2013), suggesting that improvements in the differentiation protocols are still warranted.

In most of these studies, the induced pluripotent stem cell‐derived hepatocyte‐like cells were obtained by maturation in 2D cultures, and the cells are loaded in a bioreactor only after maturation. Hepatic differentiation and maturation directly in the 3D bioreactor may offer great advantages such as overcoming the need to harvest the total amount of cells needed for the BAL from the 2D culture and loading in a 3D bioreactor. However, relatively few studies have investigated the hepatic differentiation of stem cells directly in a 3D perfusion bioreactor or BAL using embryonic stem cells (ESC; Fonsato et al., 2010; Miki, Ring, & Gerlach, 2011; Pekor, Gerlach, Nettleship, & Schmelzer, 2015; Schmelzer et al., 2010; Sivertsson, Synnergren, Jensen, Bjorquist, & Ingelman‐Sundberg, 2013; Wang et al., 2012), and only three of them used hiPSCs (Freyer et al., 2016; Giobbe et al., 2015; Luni et al., 2016). Flow of the medium was shown to have beneficial effects on hepatic differentiation of ESC and fetal liver cells and to improve liver functions of ESC‐derived hepatocytes (Lu et al., 2015; Pekor et al., 2015; Schmelzer et al., 2010; Yu et al., 2014). Even simple recirculation of medium in a rotating bioreactor improved the function of the differentiated hepatocyte‐like cells (Fonsato et al., 2010; Wang et al., 2012). The flow may not only physically influence the cells by creating flow forces, but it also may improve mass as well as gas transfer between the cells and the medium and promote the removal of waste products (Pekor et al., 2015; Yu et al., 2014). However, in another study, perfusion inhibited adipogenic differentiation of adipose‐derived stem cells possibly by washing away autocrine or paracrine factors (Hemmingsen et al., 2013).

A limitation of many studies is the absence of a proper benchmark to evaluate whether the cells are fully differentiated with respect to the expression levels of liver‐specific markers and liver functions of the generated hepatocytes. For example, many studies have not used a benchmark at all, whereas others have used PHH cultured in vitro for 2 days or more (Iwamuro et al., 2012; Song et al., 2009; Takayama, Inamura, Kawabata, Katayama, et al., 2012; Takayama, Inamura, Kawabata, Sugawara, et al., 2012). However, it is known that PHH cultured beyond 24–48 hr rapidly lose their phenotype and liver‐specific functions, and using these cells as a benchmark results therefore in an overestimation of the maturation level of the stem cell‐derived hepatocytes (Ulvestad et al., 2013). By contrast, fresh human precision‐cut liver slices (hPCLS) contain hepatocytes in their natural 3D tissue‐matrix configuration, in contact to the other liver cell types, and retain expression as well as activity of Phase I and Phase II metabolic enzymes at levels comparable with the in vivo situation (Elferink et al., 2011). Therefore, hPCLS can be considered the gold standard for assessing the maturation of stem cell‐derived hepatocytes into fully differentiated hepatocytes.

Here, we differentiate hiPSC‐derived definitive endoderm (DE) cells into hepatocytes in situ in a perfusion bioreactor system. Hepatic differentiation and functionality of hiPSC‐derived hepatocytes were assessed using fresh hPCLS as benchmark for ex vivo liver, and 2D static cultures were used to compare differentiation efficacy in 2D static and 3D flow systems.

## 2 MATERIALS AND METHODS

### 2.1 Differentiation of hiPSC‐DE cells into hepatocytes under static conditions and flow conditions

**Figure 1:**
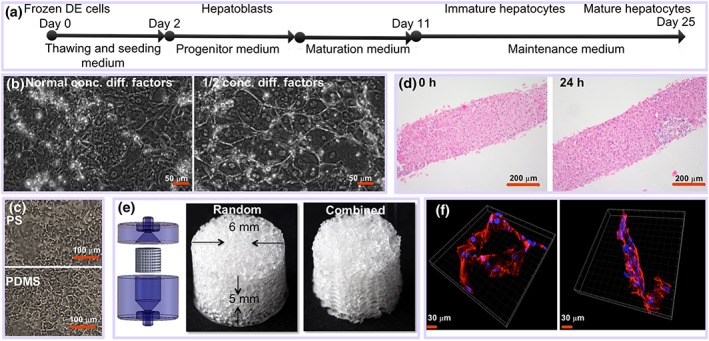
(a) Schematic overview of the experimental process of differentiation of DE cells to mature hepatocytes. (b) Differentiation of induced pluripotent stem cell (iPSC) under flow regime (500 nl/min) on flat surface for 25 days using normal concentration of differentiation factors or ½ concentration of differentiation factors. (c) Differentiation of iPSC for 19 days on polystyrene (PS) and on polydimethylsiloxane (PDMS). (d) Human precision‐cut liver slices at 0 and 24 hr of incubation (haematoxylin–eosin staining). (e) Scaffolds and house (left panel) used for perfusion. (f) Confocal microscopy images of iPSCS‐derived hepatocyte‐like cells. Blue is nucleus (DAPI), and red is actin staining [Colour figure can be viewed at http://wileyonlinelibrary.com]

Human iPS‐derived DE cells (Cellartis Definitive Endoderm ChiPSC18, Cat. No. Y10040) derived from human dermal fibroblasts, authenticated using Short Tandem Repeat (STR), and mycoplasma free according to quantitative PCR (see further information about this cell line on http://www.clontech.com) were cultured and differentiated into hepatocytes for 25 days according to the suppliers' recommendations in the Cellartis Hepatocyte Differentiation Kit (Cat. No. Y30050; see Figure 1a). Briefly, the cell culture surface (cell culture plates or scaffold) was coated with Hepatocyte Coating (from Cellartis Hepatocyte Differentiation Kit, Cat. No. Y30050) at 37 °C for 1–2 days and subsequently washed with phosphate buffered saline solution (10 mM Na phosphate in 0.9% NaCl, pH 7.4). DE cells were thawed and seeded in Hepatocyte Thawing and Seeding Medium at an initial density of 2.5 × 10^6^ cells/scaffold and for the static references 1.5 × 10^5^ cells/cm^2^ in 24‐well plate format (using polystyrene [PS] well plates or corresponding polydimethylsiloxane [PDMS]‐coated well plates, see below) in 1 ml of medium. The DE cells were differentiated in Hepatocyte Thawing and Seeding Medium for 2 days at 37 °C, before changing to Hepatocyte Progenitor Medium for another 5 days of differentiation to hepatoblasts. The cells were then differentiated further in Hepatocyte Maturation Medium for 4 days to immature hepatocytes and finally matured in Hepatocyte Maintenance Medium for another 14 days of culture to mature hepatocytes. In the static cultures, the medium was exchanged every 2–3 days. The derivation of DE cells from iPSCs as well as the robustness of the hepatic differentiation protocol has already been shown before in 2D conditions on hiPSC cell lines from different donors (Asplund et al., 2016).

### 2.2 Scaffolds fabrication and perfusion cell differentiation culture

PDMS was chosen as scaffold material instead of hydrogels because of its good biocompatibility and structural stability and possibility to scale it up enabling production of litre‐sized scaffolds (Mohanty et al., 2016). Random porous scaffolds (Figure 1e) were fabricated from PDMS by using salt leaching techniques similar to that described previously (Yuen, Su, Goral, & Fink, 2011). Hexagonal combined structured or porous scaffolds (Figure 1e) were made using a sacrificial mould with hexagonal pattern fabricated by 3D printing using commercially available water dissolvable polyvinyl alcohol (MakerBot, USA) and packed with salt crystals as described before. The scaffolds were treated with oxygen plasma (125 W, 13.5 MHz, 50 sccm, and 40 millitorr) to render their surfaces hydrophilic and sterilized by autoclaving. They were coated with Hepatocyte Coating (from Cellartis Hepatocyte Differentiation Kit, Cat. No. Y30050) by centrifugation at 300 *g* for 5 min and then left overnight at 37 °C. The scaffolds were subsequently washed with phosphate buffered saline centrifugation at 300 *g* for 5 min and then left in a medium at 37 °C for 2 hr prior to being used to experiments.

A self‐sustained perfusion system with 16 parallel reactors was constructed (Figure S1) holding PDMS scaffolds. The scaffold bioreactor array, glass vials, caps, and poly(tetrafluoroethylene) tubing were sterilized by autoclaving before assembling in a laminar flow bench. A 0.5 M NaOH was flushed throughout the system to ensure a sterile fluidic path. The system was subsequently flushed with sterile water and then with culture medium. Coated scaffolds were placed in cylindrical holes in a custom built tray. A 2.5 × 10^6^ freshly thawed DE cells in 30 μl of Hepatocyte Thawing and Seeding Medium was pipetted into each scaffold, and cells were allowed to adhere for 3 hr at 37 °C under 95% air/5% CO_2_. The seeding tray was inverted as well as placed vertically in four different positions to allow the cells to distribute throughout the scaffolds during 3 hr. The scaffolds were then placed in the 4 × 4 bioreactor array of the fluidic platform, and media were perfused through the scaffolds at flow rates of either 1 or 5 μl/min. The entire system was incubated at 37 °C under 95%air/5% CO_2_. Cells were cultured and differentiated for 25 days.

### 2.3 Human liver tissue

Human liver material was obtained from liver tissue of 10 individual patients, remaining as surgical waste after reduced liver transplantation patients, from liver tissue donated after cardiac death but not suitable for transplantation due to the age, or from patients undergoing hepatectomy for the removal of carcinoma. This study was approved by the Medical Ethical Committee of the University Medical Centre Groningen, according to Dutch legislation and the Code of Conduct for dealing responsibly with human tissue in the context of health research (http://www.federa.org/), refraining the need of written consent for “further use” of coded‐anonymous human tissue. The procedures were carried out in accordance with the experimental protocols approved by the Medical Ethical Committee of the University Medical Centre Groningen.

hPCLS were prepared as described previously by de Graaf et al. (2010). The hPCLS were made about 200 μm thick and had 5‐mg wet weight. In order to remove cell debris and to restore function, hPCLS were preincubated in the incubator (Panasonic, USA) for 1 hr at 37 °C in a 12‐well plate filled with 1.3 ml of Williams' Medium E (Gibco, USA) saturated with 80%O_2_/5%CO_2_ while gently shaking 90 cycles per minute.

#### 2.3.1 Static hPCLS culture

After preincubation, slices were transferred individually to a 12‐well plate filled with 1.3 ml of Hepatocyte Maintenance Medium (from Cellartis Hepatocyte Diff Kit; Cat. No. Y30050) saturated with 80%O_2_/5CO_2_ and supplemented with 50 μg/ml gentamycin (Invitrogen). Plates were gently shaken at a rate of 90 cycles per minute in the incubator at 37 °C.

#### 2.3.2 hPCLS culture under flow condition

After preincubation, slices were transferred individually into small micro‐chambers of PDMS biochips. The fabrication process of the biochip, as well as a schematic view of the biochip set‐up, was extensively described before (van Midwoud, Groothuis, Merema, & Verpoorte, 2010). Slices were embedded in Matrigel (BD Biosciences, Bedford, MA, USA) as described previously, and the biochips were perfused with 2 times diluted Hepatocyte Maintenance Medium from Cellartis Hepatocyte Diff Kit supplemented with 50 mg/ml gentamycin at 10 μl/min flow in a humidified incubation chamber saturated with a mixture of 95%O_2_/5%CO_2_ as described in detail before (van Midwoud, Merema, Verweij, et al., 2011). Viability of hPCLS was assessed by analysis of ATP content and morphological examination after 0 and 24 hr. More details are provided in the Supporting Information.

### 2.4 Imaging and confocal microscopy

Phase contrast images of 2D flow cultures and fluorescence‐based imaging of the scaffolds were acquired by a Zeiss Axio Observer as described in detail in the Supporting Information. Confocal acquisitions of the scaffolds were performed using a Zeiss LSM 700 module in the Axio Imager M2 upright microscope using a 40×/1.20 W Korr C‐Apo objective. More details are provided in the Supporting Information.

### 2.5 Functional characterization of hiPSC‐derived hepatocytes and hPCLS

#### 2.5.1 Phase I metabolism

To test the activities of several different CYP isoenzymes, hPCLS and cells in perfused and static systems were exposed for 1–3 hr to a drug cocktail containing 10 μM phenacetin (CYP1A), 10 μM bupropion (CYP2B6), 50 μM mephenytoin (CYP2C19), 10 μM diclofenac (CYP2C9), 10 μM bufuralol (CYP2D6), and 5 μM midazolam (CYP3A) in Hepatocyte Maintenance Medium without phenol red and supplemented with 2 mM L‐glutamine and antibiotics (50 mg/ml gentamycin for hPCLS and 0.1% penicillin and streptavidin for cells). Medium was collected and stored at −80 °C until further analysis. Metabolite concentrations were measured at Pharmacelsus (Germany) by liquid chromatography–mass spectrometry according to in house protocols. The metabolite production was normalized per milligram protein and per hour.

#### 2.5.2 Phase II metabolism

For Phase II metabolism studies, hPCLS and cells in perfused systems or in static condition were exposed to 100 μM of 7‐hydroxycoumarin (7‐HC; Sigma‐Aldrich, St.Louis, MO, USA) for 1–3 hr. Medium was collected at outlet tubes or from the incubation medium and stored at −20 °C until further analysis, using 7‐HC, 7‐HC‐glucuronide, and 7‐HC‐sulfate as standards. The metabolite production was normalized per milligram protein and per hour.

### 2.6 Gene expression

Total cellular RNA from cells or PCLS was purified by using the RNeasy Micro kit (Qiagen, 74004) or using Maxwell 16 LEV simplyRNA Tissue Kit (Promega, USA), respectively. The RNA was converted to cDNA using the High Capacity cDNA Reverse Transcription Kit (Applied Biosystems, 4374966), and quantitative real‐time PCR was conducted using the TaqMan Gene Expression Assays (Applied Biosystems 4331182). More details are provided in the Supporting Information.

### 2.7 Statistical analysis

Four independent experiments were performed with four batches of DE cells from one donor and hPCLS from seven different human donors. Because the number of donor livers for slices and number of donors for stem cells are limited, and inter‐individual variations are large in the human population, we conclude on the differentiation of the cells by comparing the range of expression or activity rather than the mean or median values.

Additional methods can be found in the Supporting Information.

## 3 RESULTS

### 3.1 Differentiation of DE cells into hepatocyte‐like cells under 3D flow condition

Differentiation of hiPSC‐derived DE cells into hepatocytes was performed in perfused 3D bioreactors with highly porous 3D PDMS scaffolds and for comparison in conventional 2D cultures in PS wells or PDMS‐coated wells. Frozen DE cells were seeded at a density of 2.5 × 10^6^ cells per scaffold and differentiated into hepatocytes as illustrated in Figure 1a. Differentiation under flow on 2D surface showed very good morphology of differentiated cells (Figure 1b), provided that the amount of differentiation factors was reduced by 50%, because 100% differentiation factor concentration results in poor cell adhesion during flow (Figure S2, left panels). Cells differentiated on PS and PDMS, respectively, under static conditions showed similar morphology (Figure 1c; Figure S3). It was not possible to obtain bright field images of the cells in the 3D scaffold due to the poor optical characteristics of the scaffold.

The cells adhered typically in clusters in the scaffolds (Figure 1f) at a relatively low overall final cell density (200.000–300.000 cells/scaffold) as determined by visual inspection (Figure S4) and measurement of protein content (Figure S5) in the scaffold. As DE cells and differentiated hepatocytes adhere well to PDMS (Figure S4), the relatively low number of adhering cells to the scaffold is likely due to difficulties to seed the cells in the scaffolds, although seeding was performed by rotating the scaffolds in six different directions with 30‐min incubation in each direction. Calculations indicate that a scaffold has a surface area of approximately 10 cm^2^ (Figure S6), and thus, the cells in the 3D scaffold had an effective cell density of 20,000–30,000 cells/cm^2^, which is lower than the corresponding 2D static cultures (usually 80,000–100,000 stem cell‐derived hepatocytes/cm^2^; data not shown). However, as determined by visual inspection, the cells adhered typically in clusters, and therefore, the local cell densities were probably higher.

Differentiation of hiPSC‐derived DE cells loaded in the scaffolds was performed at a flow rate of 1 μl/min (exchange rate every 50 min) and 5 μl/min (exchange rate every 10 min). It was calculated that in both flow regimens, shear forces were very low (1.1e‐4 dyn/cm^2^ or less; Figures S7 and S8). Furthermore, the exchange rate of once per 10 min was shown to be compatible with differentiation into hepatocytes in 2D flow cultures (Figure S2) and has previously shown to support differentiation of adipose‐derived stem cells into adipocytes using conditioned medium (Hemmingsen et al., 2013).

### 3.2 Comparison of gene expression of hepatocyte markers of human iPSC‐derived hepatocytes and hPCLS

We investigated the hepatic differentiation of hiPSC‐derived DE cells in the 3D scaffold at two different flow rates, 1 and 5 μl/min, and in two different scaffold designs (Figure 1e) and compared the results with cells differentiated under static 2D conditions in standard PS well plates or in well plates coated with a PDMS layer. hPCLS were used as a benchmark for cells differentiated in the scaffold under perfusion. hPCLS were prepared from 10 individual livers of human donors, aged 20–73 years (60% female) as described (de Graaf et al., 2010). Due to the limited amount of liver material, not every test was performed on all donor livers. The morphological appearance and ATP content of the hPCLS after a preincubation of 1 hr to restore ATP levels (0 hr) and after 24 hr of incubation indicated that the slices were viable. ATP levels were 9.7 ± 1.3 and 8.24 ± 0.76 pmol/μg protein at 0 and 24 hr, respectively (mean ± standard error of the mean). Morphology showed intact liver tissue at 0 and after 24 hr of incubation (Figure 1d).

**Figure 2:**
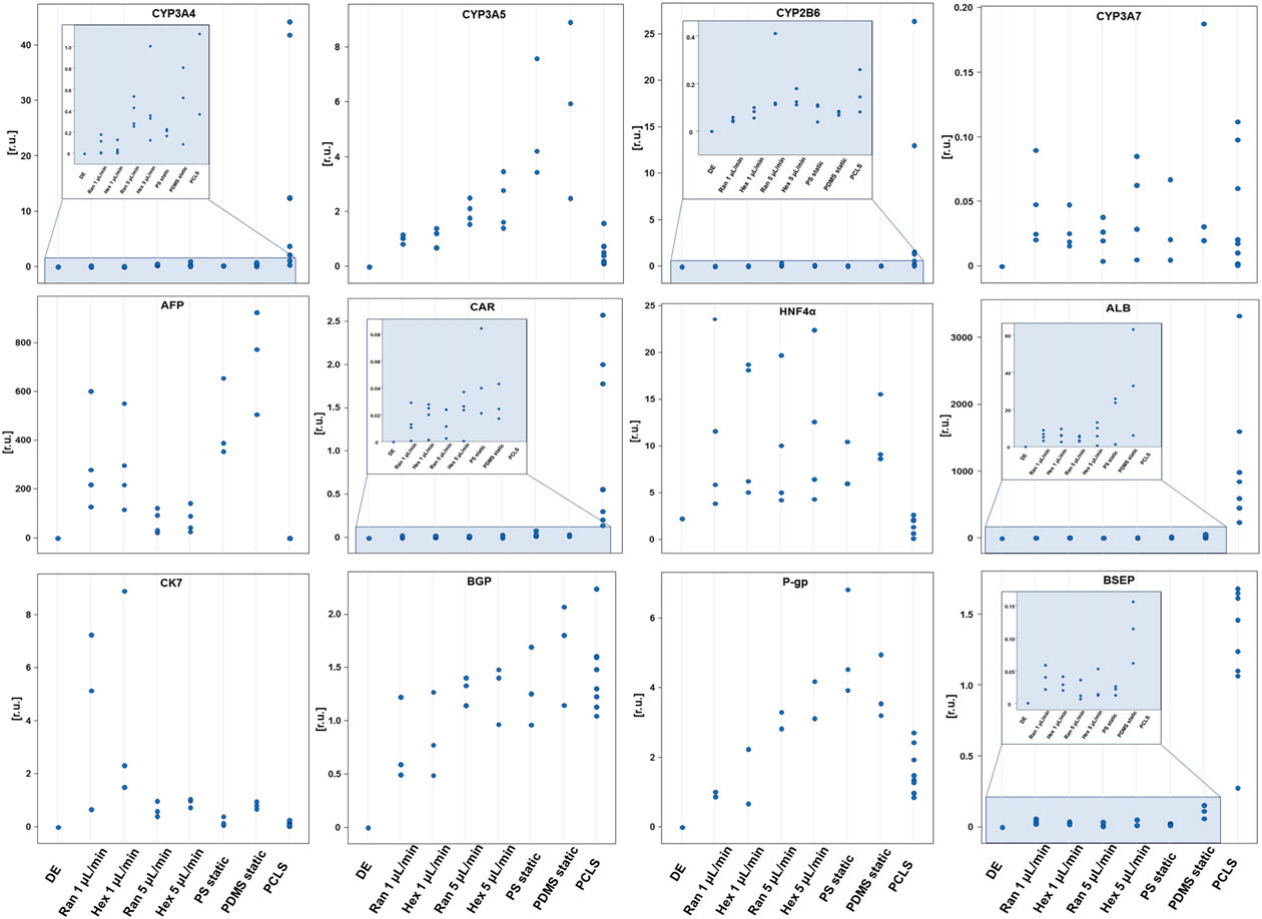
Expression of different hepatic genes by definitive endoderm (DE) cells cultured and differentiated under different conditions. Data are given for each individual sample to appreciate the variation within each condition and the overlap between the different conditions. Data are presented as Ct values of the respective genes normalized to Ct values of the housekeeping gene CREBBP. Results are from four independent differentiation experiments and seven donors. Due to poor RNA yield, some genes where only analysed in two (P‐gp) or three (CK7, BSEP, and BGP) of the cultures. PCLS = precision‐cut liver slices; PDMS = polydimethylsiloxane; PS = polystyrene [Colour figure can be viewed at http://wileyonlinelibrary.com]

**Figure 3:**
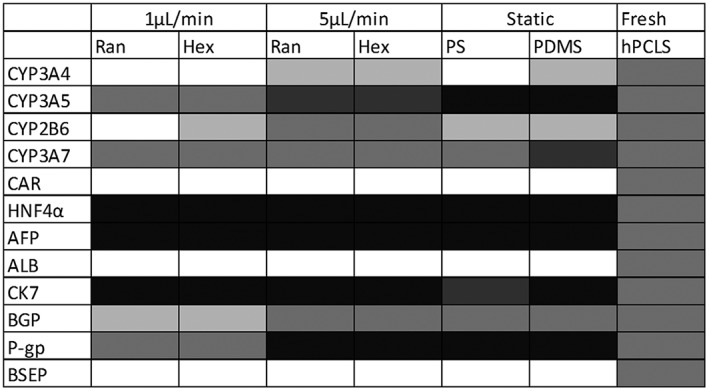
Summary of the comparison of the gene expression levels between the differentiated cells and the human precision‐cut liver slices (hPCLS) as benchmark from Figure 3. The different grades of colour in the chart represent the gene expression levels in the human‐induced pluripotent stem cells‐derived hepatocytes relative to the gene expression levels in hPCLS as follows: Black = all individual data of the cells are higher than those in hPCLS; dark grey = the individual data of the cells are higher than or in the higher range of those of hPCLS; Middle grey = all data of the cells are in the same range as those of hPCLS; light grey = the individual data of the cells are lower than or in the lower range of those of hPCLS; white = all individual data of the cells are lower than those of the hPCLS. PDMS = polydimethylsiloxane; PS = polystyrene

The gene expression of the liver‐specific genes of the cells differentiated under the conditions outlined above and the hPCLS is depicted in Figure 2. A summary based on classification of gene expression into broader groups is presented in Figure 3. When comparing the differentiated cells in static cultures on PS with hPCLS, most of the CYP genes, the expression of the epithelial biliary cell markers (CK7 and BGP), and the drug transporter ABCB1 (multidrug resistance protein, P‐gp) in the cells were in the range of that seen in hPCLS. Large differences in gene expression were observed for the genes CAR, ALB, and BSEP. These genes were clearly expressed lower in differentiated cells compared with hPCLS. These three genes were, however, expressed higher in differentiated cells than in the DE cells (Figures 2 and 3). Furthermore, the differentiated cells showed higher expression of AFP and HNF4a than the hPCLS. The differentiated cells on PS therefore had a mixed phenotype, where some genes suggest a partly to fully maturated phenotype (HNF4a, CYP3A4, 3A5, 3A7, and 2B6 and P‐gp), whereas others suggest a less mature phenotype (ALB, AFP, CAR, and BSEP). In addition, maturation of a part of the cells into biliary epithelial cells is suggested by expression of CK7 and BGP.

Differentiation on the scaffold material PDMS in static cultures had only a minor impact on the gene expression compared with cells on PS; all genes showed overlapping expression in cells on PDMS and PS, respectively (Figures 2 and 3).

Flow modulated the gene expression of differentiated cells only to a small extent, with 5 μl/min performing slightly better than 1 μl/min for CYP 3A4 and 2B6. Flow also modulated the ALB and AFP expression; the ALB expression was suppressed by flow compared with the corresponding static cultures. The AFP expression was lowest in cells exposed to the 5 μl/min perfusion compared with static and perfusion with 1 μl/min but did not result in the very low levels observed in hPCLS (Figures 2 and 3).

### 3.3 Liver functions

Phase I and Phase II metabolic activities, albumin, urea, and total bile acid production were analysed in hPCLS and the hiPSC‐derived hepatocytes for each of the different conditions described above.

#### 3.3.1 Phase I metabolism

Differentiated hiPSC‐derived DE cells as well as hPCLS were exposed to the substrates under both static and flow conditions to account for possible effects of flow on metabolism. The metabolic activities in the hPCLS showed large inter‐donor variations as expected, as inter‐individual differences in drug metabolism are well described. On the other hand, the source of the human liver tissue or age of the patients (aged from 20 to 73 years) did not have an effect on and did not correlate with the functional capacities of the liver.

**Figure 4:**
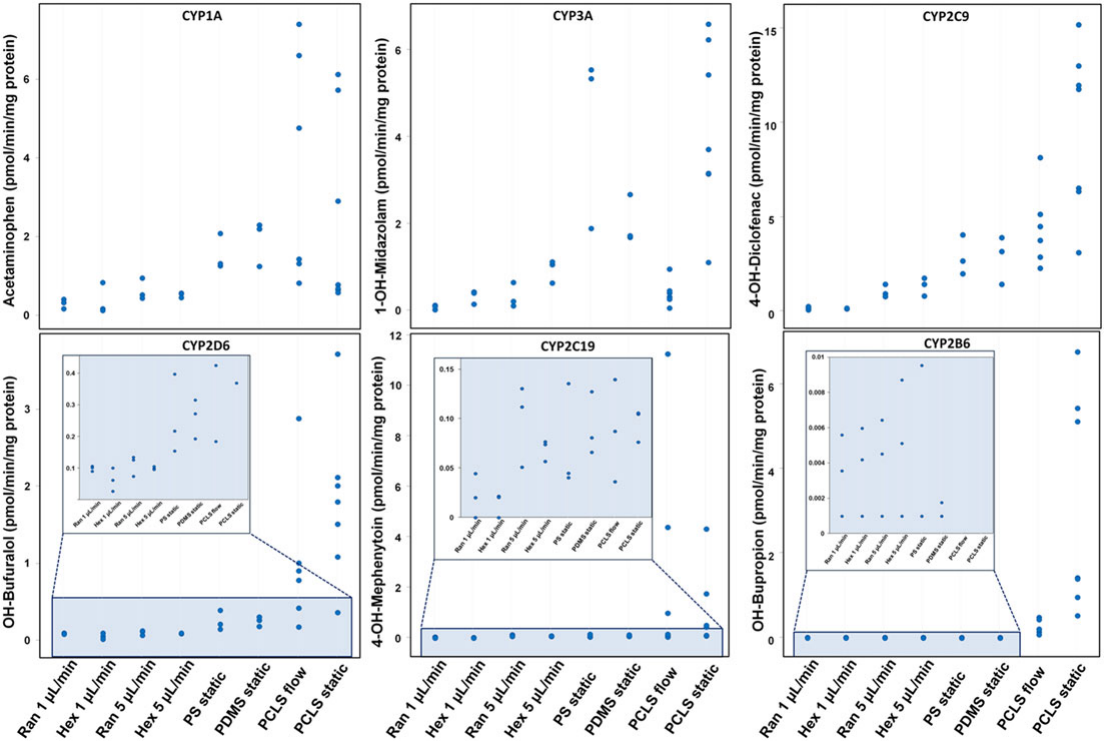
Phase I metabolite production of midazolam, phenacetin, diclofenac, bufuralol, bupropion, and mephenytoin by human‐induced pluripotent stem cells‐derived hepatocytes and human precision‐cut liver slices cultured in static or under flow conditions. The individual values are expressed as pmol·min^−1^·mg^−1^ protein. Results are from three independent differentiation experiments and seven donors. PCLS = precision‐cut liver slices; PDMS = polydimethylsiloxane; PS = polystyrene [Colour figure can be viewed at http://wileyonlinelibrary.com]

Overall, the hPCLS showed similar metabolic activity when cultured under flow or in static conditions, although a lower metabolic activity was found for CYP3A and CYP2B6 activity under flow (Figure 4). These differences may be explained by binding of the lipophilic substrates midazolam and bupropion to the PDMS of the biochip (van Midwoud, Merema, Verpoorte, et al., 2011), although it cannot be excluded that the perfusion conditions may have influenced the metabolic activity.

The hiPSC‐derived hepatocytes differentiated under flow conditions as well as under static conditions showed overlapping activities of CYP3A, CYP1A, CYP2C9, CYP2D6, and CYP2C19 with the hPCLS (Figure 4). However, at a flow of 1 μl/min, somewhat lower activities of CYP2C9 and CYP2C19 were found compared with a flow of 5 μl/min. CYP2B6 showed very low activities in cells compared with hPCLS irrespective of perfusion or static culture conditions. The overlap in activities of most of the CYP isoforms, with exception of CYP2B6, in the differentiated cells with those in hPCLS under flow indicates a high degree of hepatocyte drug metabolic function in the differentiated cells.

#### 3.3.2 Phase II metabolism

**Figure 5:**
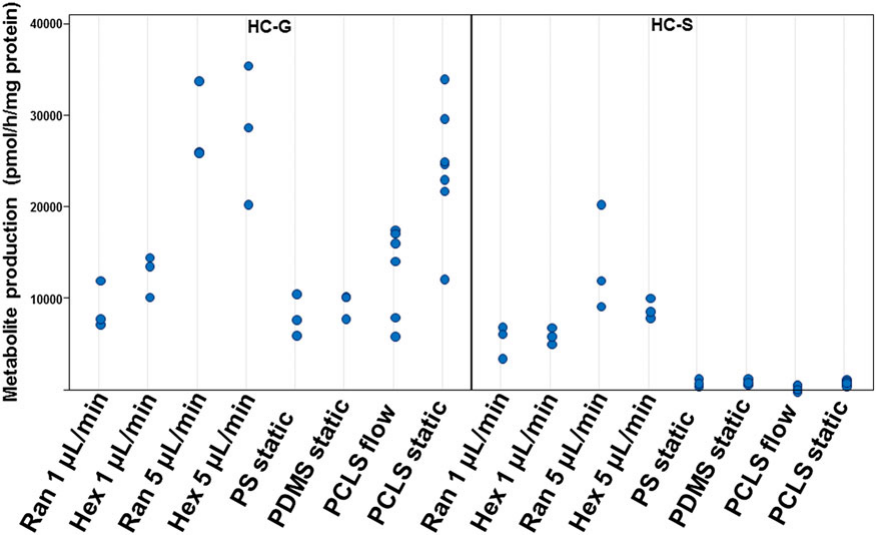
Production of 7‐hydroxycoumarin glucuronide (HC‐G; left panel) and 7‐hydrocycoumarine sulfate (HC‐S; right panel) from 7‐hydroxycoumarin by human‐induced pluripotent stem cells‐derived hepatocytes and human precision‐cut liver slices cultured in static or under flow conditions. The individual values are expressed as pmol·hr^−1^·mg^−1^ protein. Results are from three independent differentiation experiments and seven donors. PCLS = precision‐cut liver slices; PDMS = polydimethylsiloxane; PS = polystyrene [Colour figure can be viewed at http://wileyonlinelibrary.com]

Differentiated cells exhibited high uridine UDP‐glucuronyltransferase and sulfotransferase activities when exposed to 7‐HC (Figure 5). Both Phase II activities were higher in hiPSC‐derived hepatocytes at a flow of 5 μl/min than at 1 μl/min. Although the activities in cells cultured under static conditions were similar to those in hPCLS static cultures, the 7‐HC‐glucuronide production by cells cultured at 5 μl/min flow in both hexagonal and random scaffolds was on average twofold higher than in liver slices at flow conditions. In addition, the sulfation rate of 7‐HC resulting in 7‐HC‐sulfate was 30–40‐fold higher in cells differentiated under flow condition compared with differentiation under static condition and the hPCLS.

#### 3.3.3 Albumin production

**Figure 6:**
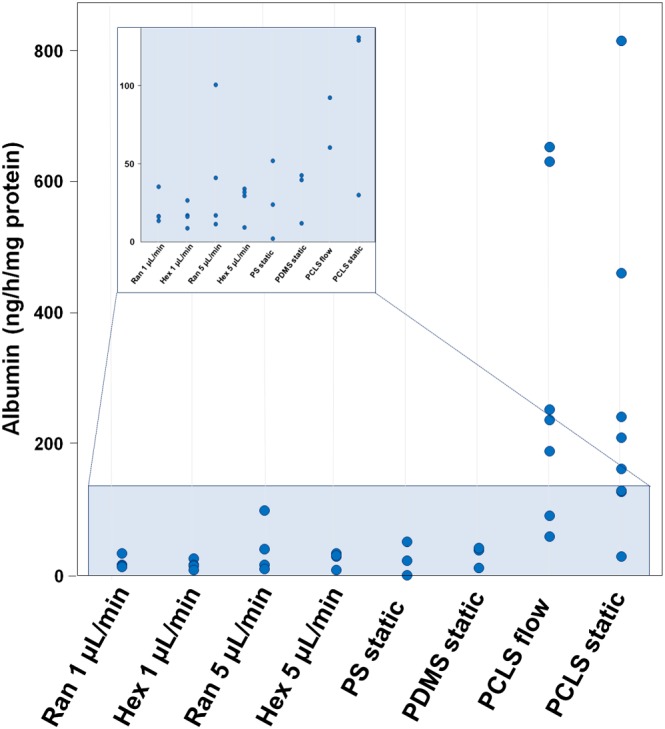
Albumin production by human‐induced pluripotent stem cells‐derived hepatocytes and human precision‐cut liver slices cultured in static or under flow conditions. The individual data values are expressed as ng/hr/mg protein. Results are from three independent of differentiation experiments and seven donors. PCLS = precision‐cut liver slices; PDMS = polydimethylsiloxane; PS = polystyrene [Colour figure can be viewed at http://wileyonlinelibrary.com]

Albumin production by the hiPSC‐derived hepatocytes was overlapping with the lower range of that of hPCLS (Figure 6). No difference was observed between the two types of scaffolds at both flow rates or between static and perfusion cultures.

#### 3.3.4 Bile acid secretion and urea synthesis

Bile acid secretion by the hiPSC‐derived hepatocytes was at the same level of 25–30 pmol·hr^−1^·mg^−1^ protein in cells differentiated under static conditions for 22 or 24 days as in hPCLS (Figure S9). We could not detect bile acids in the samples of the outflow medium obtained of the scaffolds. This was probably because the excreted compounds were highly diluted by the perfusion flow rate. The observed total bile acid secretion of 25–30 pmol·hr^−1^·mg^−1^ protein by differentiated cells or hPCLS would result in a concentration of about 20–100 pmol/ml at a flow rate of 1 and 5 μl/min, respectively, in the BAL, which is below the detection limit of the bile acid assay.

On average, the urea production by hiPSC‐derived hepatocytes was below or in the lower range of that of hPCLS (0.06–7.6 μg·hr^−1^·mg^−1^ protein for hiPSC‐derived hepatocytes and 1.6–11.9 μg·hr^−1^·mg^−1^ protein for hPCLS; Figure S10). Cells differentiated at 5 μl/min flow and under static conditions tended to show higher urea synthesis (0.3–7.6 μg·hr^−1^·mg^−1^ protein) than those differentiated under 1 μl/min flow (0.06–0.55 μg·hr^−1^·mg^−1^ protein).

## 4 DISCUSSION

We have obtained highly differentiated hepatocytes from hiPSCs under flow conditions in a BAL. To assess their differentiation status, we compared the expression and function of the cells in this BAL model with fresh human liver slices that have in vivo‐like activities (de Graaf et al., 2010; van Midwoud, Merema, Verpoorte, et al., 2011) and found as yet unprecedented liver functions in the differentiated cells. Moreover, we found that DE cells can be successfully differentiated into hepatocyte‐like cells in a 3D scaffold in a bioreactor under flow conditions, to a similar or only slightly better differentiation grade than under static 2D conditions, especially with respect to Phase II sulfation activity and a lower AFP expression, which can make the production of a BAL easier and more effective in the future.

hiPSC‐derived cells differentiated under flow in a 3D bioreactor resulted in a BAL model with overlapping Phase I metabolism (except for CYP2B6) and similar or higher Phase II metabolism, compared with fresh human liver slices. Urea production was present in the hiPSC but was below or in the lower range of the hPCLS. However, the capacity of urea production in the BAL from externally added ammonia, in order to mimic ammonia from extrahepatic tissues, was not assessed because no ammonia was added to the medium. Further studies with exposure to extracellular ammonia are needed to show the ability of the cells to detoxify ammonia, which is important for patients with liver diseases where high concentrations of neurotoxic ammonia are detected. Bile acids were produced by hiPSC‐derived hepatocytes at the same rate as in fresh tissue slices. The gene expression of P‐gp in hiPSC‐derived hepatocytes was shown to be higher than in hPCLS, which is remarkable as a 10–20‐fold lower expression was found in differentiated hiPSC compared with human hepatocytes by Lu et al. (2015). However, the gene expression of BSEP in the differentiated cells was lower than in the hPCLS. The hiPSC‐derived cells also expressed both CK‐7 and BGP, similar to the hPCLS, indicating that in addition to the hiPSC‐derived hepatocytes, also some biliary epithelial cells are present. This bipotent differentiation potential of iPSC‐derived hepatic progenitor cells was also found previously (Freyer et al., 2016; Yanagida, Ito, Chikada, Nakauchi, & Kamiya, 2013). The albumin secretion of stem cell‐derived hepatocytes achieved here is similar to hiPSC‐derived hepatocytes found by Gieseck et al. (2014) and 10–100‐fold higher than in human ESC‐derived hepatocytes (Miki et al., 2011) and 3–40‐fold higher than mouse iPSC‐derived hepatocytes (Iwamuro et al., 2012) but lower than in fresh tissue slices. Although the mRNA expression was high for HNF4a indicating hepatic differentiation and low for CYP3A7, which is a fetal enzyme with low expression in the adult liver, the relatively high expression of the fetal protein AFP indicates that maturation of the cells is not fully complete. This has also been observed by others (Chen et al., 2012; Freyer et al., 2016; Gieseck et al., 2014; Iwamuro et al., 2012; Kim et al., 2015; Sasaki et al., 2013). It still needs to be addressed how relevant this is for the functioning of the BAL in patients who need liver support or for toxicity testing. Taken together, these results show that hiPSC differentiated under static conditions as well as under flow in a scaffold have liver functions close to those in fresh human liver tissue. The significant improvements with respect to liver functions of the differentiated cells presented here compared with other studies could be due to better differentiation protocols, whereas the difference between the cells differentiated under 2D static and 3D perfusion conditions could be ascribed to better nutrient delivery and waste removal.

Most studies have used PHH cultured in vitro for 1–3 days as benchmark for hepatic activity (Gieseck et al., 2014; Lu et al., 2015; Takayama, Inamura, Kawabata, Katayama, et al., 2012; Takayama, Inamura, Kawabata, Sugawara, et al., 2012; Ulvestad et al., 2013). Because PHH functions decrease rapidly and drastically (10–1000‐fold after 48 hr culture) during in vitro culture (Ulvestad et al., 2013), the use of these PHH as standard tends to overestimate the metabolic function of hiPSC‐derived hepatocytes. Therefore, the comparison of those data with our study is difficult. Moreover, comparison of the metabolic activity data between different studies is further hampered by the fact that the substrate concentrations and experimental conditions used are largely different. Fresh human PCLS, on the other hand, show similar metabolic activity as the fresh PHH and give a good representation of liver functions in vivo (Asplund et al., 2016). Even though nowadays it is possible to culture PHH for several days with preservation of their metabolic capacities in certain culture conditions, none of the above‐mentioned studies have shown that the appropriate measures have been taken to maintain PHH functions. The only three published studies that used fresh hepatocytes or hepatocytes cultured for maximally 24 hr as a control are the study of Ulvestad et al. (2013), Freyer et al. (2016), and Meier et al. (2017). Comparison of the results of those studies with our data shows that the CYP3A, CYP2C9, and CYP1A activities of hiPSC‐derived hepatocytes in our study were 10 to several hundred folds higher. In the study of Freyer et al., no CYP2C9 activity at all was detected in the derived hepatocytes. We were the first to measure Phase II metabolism in hiPSC and found that glucuronidation was comparable with PCLS, but sulfation was remarkably higher after differentiation under flow, which requires further studies.

The gene expression of CYP‐enzymes and their activity varied notably between donor livers. This variation is very well known in the human population, which is among others a result of polymorphisms and induction by environmental and physiological factors. For example, CYP1A2, CYP2D6, CYP2C9, CYP2C19, CYP2B6, and CYP3A4 are known to be polymorphic and highly inducible enzymes in human (Zhou, Liu, & Chowbay, 2009). With this in mind, it is noteworthy that the gene expression and enzyme activities in the hiPSC‐derived cells overlapped in most cases with those in the hPCLS, with a few discrepancies noted below. For example, the CYP3A5 gene was higher expressed in hiPSC‐derived hepatocytes compared with hPCLS, whereas CYP3A4 gene expression only reached up to the lower range of human livers (Figure 2). The fact that CYP3A4 and 3A5 have strongly overlapping substrate specificities (Emoto & Iwasaki, 2006) may explain why the total CYP3A metabolism of midazolam was similar in hiPSC‐derived hepatocytes and hPCLS (Figure 4). Although the gene expression of CYP2B6 in the differentiated cells was in the lower range of hPCLS, possibly due to low CAR expression, the activity of this enzyme was at least 10 times lower in hiPSC‐derived hepatocytes than in hPCLS indicating a post‐transcriptional regulation. Future research will be focused to improve the as yet under‐expressed CAR‐mediated pathway.

We found a limited influence of the flow rate on the hepatic differentiation of hiPSC in the BAL. A flow of 5 μl/min resulted in a slightly better hepatocyte differentiation and maturation than the 1 μl/min flow. This might be explained by the better nutrient and oxygen supply and removal of waste metabolites at the higher flow rate. Also, the type of scaffold had no obvious impact on the differentiation.

In conclusion, most of the drug metabolism enzyme activities of the developed hiPSC‐based BAL were in the same order of magnitude as in the fresh human tissue, which is an important achievement in liver tissue engineering and is thus promising for future applications in drug metabolism and toxicity testing. A limitation of this study is that besides hepatocytes and biliary epithelial cells, which were present in the developed BAL according to gene expression profiling, no non‐parenchymal cells are present yet. Inclusion of non‐parenchymal liver cells is essential in the development of a fully functional BAL and will be a future step in this field. Also, for future toxicity tests, it is necessary to add these other liver cell types, as it is well known that these non‐parenchymal cells play an important role in drug‐induced liver injury. Moreover, future experiments with more donor individuals for both iPSC and PCLS will help to better estimate the variation in the population as well as the robustness of the differentiation protocol. Finally, future studies should show the BAL's detoxification capacities.

## CONFLICT OF INTEREST

Anders Aspegren is an employee of Takara Bio Europe.

## Supporting information


**Figure S1.** **System description.** A: Parts of the 3D perfusion system. B: Render of assembled 3D perfusion system. C: Picture of a perfusion system with tubes and vials.
**Figure S2. Effect of concentration of signaling factors on cell morphology at flow conditions.** Left panels: low magnifications, right panels high magnifications. DE cells were cultured and differentiated at 2D flow conditions in a chamber having dimensions of 1.5 mm (*w*) × 6 mm (*l*) × 0.5 mm (*h*). The concentration of signaling factors was either the normal used and optimized for static culture conditions or ½ the normal concentration but otherwise a full base medium. The cells were perfused at two different flow rates, 250 nL/min or 500 nL/min, corresponding to an exchange of the medium in the entire chamber every 20 and 10 minutes. Phase contrast images acquired at day 25. The shown images are a representative area of one chamber out of 4 chambers for each condition. A better cell attachment and less dead/floating cells were observed at perfusion with medium supplemented with half of the normal concentration of signaling factors compared to perfusion with medium supplemented with the normal concentration of signaling factors.
**Figure S3. Cell morphology of differentiating cells at static conditions.** Phase contrast images were acquired at day 9, 15, and 19. The cell morphology was very similar between the PDMS scaffold material and the PS conventional surface substrate, although some larger cells were observed on PDMS)
**Figure S4.** Microscopy imaging of hiPSC‐derived DE cells cultured and hepatic differentiated inside a porous scaffold at perfusion conditions: Image at day 22 after cell seeding of the middle of a cross‐sectioned scaffold. A) Scan of entire cross‐sectioned scaffold showing cell distribution. Höechst stained cell nuclei in blue color. B) Close‐up view of cell distribution with Höechst stained cell nuclei. The homogenous blue fields in the image are likely not cells but reflections within the scaffold. The fluorescence image is merged with a phase contrast image of the scaffold. C) Calcein‐AM live‐stained cells in green.
**Figure S5. Determination of protein content in the BAL.** DE cells were loaded into the BAL as described in material and methods. Three replicates of the four investigated BALs (two different scaffold designs and two different flow conditions), where prepared and cultured for one week. Scaffolds with cells were perfused with Dulbecco's Phosphate Buffered Saline with MgCl_2_ and CaCl_2_ (Sigma D8662) for 30 min to remove protein containing medium and then transferred to an 1.5 mL Eppendorf tube with 0.5 mL 0.2 M NaOH. To enhance distribution of NaOH within the scaffold, the tubes were vortexed for 30 seconds three times with 10 minutes incubation between each vortex. The tubes were incubated over night at 4°C. To enhance the release of cell material from the scaffold into the NaOH, the tube was again vortexed for 3 X 30 seconds with 10 minutes incubation between each vortex, and then centrifuged at 500 x g for 5 minutes. The supernatant was diluted 1:1 with milliQ water to 0.1 M NaOH. The protein content was measured by the use of the Pierce BCA Protein Assay Kit (cat. no. 23227) according to the supplier's microplate procedure. The absorbance was read at 570 nm and protein calculated based on a standard curve for bovine serum albumin. The results showed that the variance between the same type of scaffold was limited. If it is assumed that 0.1 mg protein corresponds to 100.000 cells (Anders Aspegren, personal observations), each BAL contains about 200.000–270.000 cells. This corresponds well with theoretical calculations that there can be a maximum of about 600,000–1,000,000 cells per BAL (see Supplementary Figure S6).
**Figure S6.** Theoretical calculations of the surface area of a scaffold. Two estimates were made: **Estimate 1** was based on an idealized network of cubes that are connected and then surrounded by PDMS. The calculation involved trying to find out how many salt cubes that can fit into the volume of the scaffold and then calculate the surface area by taking the number of salt particles multiplied by the total surface of one salt particle. The side of a salt cube is approximately 0.35 mm as determined by scanning electron microscopy investigations. The distance to the next salt particle is estimated to be 0.1 mm (can also be larger) meaning that a salt crystal takes up about 0.45*0.45*0.45 = 0.091125 mm^3^ including the surrounding PDMS. The cylinder volume (Figure 1C) is r^2^*pi*h = 3*3*pi*5 = 141 mm^3^. 141/0.091125 = 1556 particles. The surface area of a salt particle is 0.35*0.35*6 = 0.735 mm^2^. 1556*0.735 = 11.39 cm^2^. However because the salt crystals need to touch each other in order to form a network, some of the area is lost. In the idealized situation, each cube loses about 1.5 sides in surface area as it shares that area with other salt crystals. Therefore the area is estimated to be 11.39*9/12 = 8.5 cm^2^ In **Estimate 2** we used the measured porosity (determined to be 65%) of a random scaffold as input parameter and calculated the number of salt molecules that could fit into pores with a total volume of 0.65 multiplied by the scaffold volume: r^2^*pi*h*0.65 = 3*3*pi*5*0.65 = 92 mm^3^. Volume of salt particle is 0.35*0.35*0.35mm^3^ = 0.042875 mm^3^. Number of salt particles in scaffold are 92/0.042875 = 2145 particles. Number of particles multiplied by the surface of each salt cube (6*0.35*0.35mm^2^ = 0.735 mm^2^) = total surface volume: 1577 mm^2^ = 15.8 cm^2^. However, just as in the case above, some of the sides of the cubes are shared between each sugar cube. Using the estimate above, it is suggested that the surface area is 15.8 cm^2^*9/12 = 11.85 cm^2^. In this case, the surface area is estimated to be about 12 cm^2^, which is close to **Estimate 1** of 11.39 cm^2^. For simplicity we estimate the surface of a scaffold to 10 cm^2^ (1000 mm^2^).
**Figure S7.** Evaluation of shear stress acting on the 3D scaffold at the microscale. Velocity profile (a) and pressure gradient (b) within the bioreactor as well as in the 3D scaffold. Different COMSOL simulations were performed to calculate the shear stress that cells sense within the 3D scaffold. The first analysis was made at the macro scale, evaluating the velocity field and pressure gradient in the reactor as well as in the 3D porous scaffold.
**Figure S8.** Velocity profile (a) and shear stress (b) profile inside of a single channel having a pore diameter of 200 μm within the 3D porous scaffold. The model shows that with a pore diameter of 200 μm the shear stress acting on the wall is 1.2·10^−5^ N·m^−2^, which is beyond the shear stress limit that leads hepatocytes to death. To evaluate the magnitude of the shear stress acting on the walls of the porous channels within the 3D scaffold, an analysis at the micro‐scale was assessed.
**Figure S9. Bile acid production by hiPSC‐derived hepatocytes and human PCLS.** Graph represents mean values ± SEM. Total bile acid (TBA) content was measured using the Total Bile Acid kit (Diazyme Laboratories, Poway, CA, USA) in medium after 24 h incubation (static cultures) or 24 h perfusion (perfused cells and slices). 1 ml of medium was concentrated 10 times using the CentriVap Benchtop Vacuum Concentrator at 35°C (Labconco, Kansas City, MO, USA). TBA content was determined according to the manufacturer's protocol of the TBA kit with a few modifications. Conjugated cholic acid (50 μM) was used as a calibrator. Measurement was performed at 37°C in a 96‐well plate in the Synergy HT plate reader (BioTek, Winooski, VT, USA). The absorbance was read at 405 nm at 5 and 30 min. The TBA production is expressed as median with interquartile range.
**Figure S10. Urea production.** Urea concentrations in the medium were measured using the Urea Assay Kit (Abnova, Taiwan). Medium samples from differentiated cells and PCLS flow and static cultures were concentrated 10 times before measurement using the CentriVap Benchtop Vacuum Concentrator at 35°C. Urea content was determined according to the manufacturer's protocol of the Urea Assay kit with a few modifications. Accordingly, 25 μL of samples were added to each well and incubated for 30 min at room temperature with a reagent mix. The absorbance was read at 430 nm and urea levels calculated based on standard curve of urea standard provided with the kit and expressed as μg urea produced per h, per mg total protein. Data are expressed as individual values.
