# Supplementary material for: Differentiation of human‐induced pluripotent stem cell under flow conditions to mature hepatocytes for liver tissue engineering
Source: J Tissue Eng Regen Med. 2018 Apr 6;12(5):1273–84. doi: 10.1002/term.2659 (PMC5969064; doi:10.1002/term.2659)
Supplement: Supplementary file 1 — Figure S1. System description. A: Parts of the 3D perfusion system. B: Render of assembled 3D perfusion system. C: Picture of a perfusion system with tubes and vials. Figure S2. Effect of concentration of signaling factors on cell morphology at flow conditions. Left panels: low magnifications, right panels high magnifications. DE cells were cultured and differentiated at 2D flow conditions in a chamber having dimensions of 1.5 mm (w) × 6 mm (l) × 0.5 mm (h). The concentration of signaling factors was either the normal used and optimized for static culture conditions or ½ the normal concentration but otherwise a full base medium. The cells were perfused at two different flow rates, 250 nL/min or 500 nL/min, corresponding to an exchange of the medium in the entire chamber every 20 and 10 minutes. Phase contrast images acquired at day 25. The shown images are a representative area of one chamber out of 4 chambers for each condition. A better cell attachment and less dead/floating cells were observed at perfusion with medium supplemented with half of the normal concentration of signaling factors compared to perfusion with medium supplemented with the normal concentration of signaling factors. Figure S3. Cell morphology of differentiating cells at static conditions. Phase contrast images were acquired at day 9, 15, and 19. The cell morphology was very similar between the PDMS scaffold material and the PS conventional surface substrate, although some larger cells were observed on PDMS) Figure S4. Microscopy imaging of hiPSC‐derived DE cells cultured and hepatic differentiated inside a porous scaffold at perfusion conditions: Image at day 22 after cell seeding of the middle of a cross‐sectioned scaffold. A) Scan of entire cross‐sectioned scaffold showing cell distribution. Höechst stained cell nuclei in blue color. B) Close‐up view of cell distribution with Höechst stained cell nuclei. The homogenous blue fields in the image are likely not cells but reflections within the [file TERM-12-1273-s001.docx]

**Differentiation of Human Induced Pluripotent Stem Cell under Flow Conditions to Mature Hepatocytes for Liver Tissue Engineering**

Viktoriia Starokozhko^2*^, Mette Hemmingsen^1*^, Layla Larsen^1^, Soumyaranjan Mohanty^1^, Marjolijn Merema^2^, Rodrigo Pimentel C.^1^, Anders Wolff^1^, Jenny Emnéus^1^, Anders Aspegren^3^, Geny Groothuis^2#^, and Martin Dufva^1#^

^*^ shared first author

^#^ shared last author

**Supplementary information**

**Description of perfusion system**

A schematic view of the perfusion culture system and fluidic/air circuit is illustrated in Supplementary Figure S1. The main components of the system include an array of 4×4 bioreactors for culture of cells, vials and vial trays for storage of culture media and waste and peristaltic pumps and motors for perfusion of media. All parts are secured onto a single platform. Four 8-channel micropumps (previously described (Skafte-Pedersen *et al.*, 2009,)) that generate pulsatile flow are included in the system, controlled by motors and controllers from the Lego Mindstorm (Lego, Billund, Denmark) kit. This allows culturing of cells at four different flow rates in a single experiment. The pumps allow flow rates of sub-µl/min to approximately 90 µl/min. The fluidic circuit is formed by the pumps, bioreactor array and media storage vials that are connected using polytetrafluoroethylene (PTFE) tubing (inner diameter of 0.8 mm) (BOLA 1818-10, Bohlender GmbH, Germany). Inlet and outlet vials are coupled with PTFE tubing and supplied with air supplemented with 5% CO2 through a sterile filter. To avoid the formation of gas bubbles in the microfluidic network, an overpressure of 30 kPa is put on the flow system during its operation. All components are mounted onto a base platform for portability and user-friendly handling. The entire system can be placed in an incubator for cell culture experiments.

**Design of the bioreactor array**

The bioreactor array (Supplementary Figure S1) allows culture of 16 cylindrical 3D constructs having thicknesses of 5 mm and diameters of 6 mm (Figure 1E). The bioreactors are designed with conical inlets and outlets for uniform delivery of media through the pores of a scaffold in the cylindrical cavity (Patrachari *et al.*, 2012). Additionally, the conical inlet geometry gives rise to lower shear stress in the areas of the scaffold close to the inlet as assessed from a finite element study of the flow profile within two bioreactor designs as discussed in following sections. Silicone tubes (having inner diameter of 1.8 mm and 1 cm in height) are press fit to the ports of the bioreactor (inner diameter of 1 mm and outer diameter of 2 mm) and serve as connectors between the bioreactor and the rest of the perfusion network.

The 4×4 bioreactor array was implemented in an easily exchangeable single device, as shown in Figure S1. The upper plate of the device (having dimensions of 100 x 100 x 5 mm^3^) incorporates the outlet ports for waste removal from each bioreactor. The lower plate (with dimensions of 100 x 100 x 10 mm^3^) incorporates 4×4 cylindrical chambers (each with a diameter of 6 mm and height of 5 mm) for housing scaffolds and inlet ports for perfusion of media into the chambers. The two parts of the array are secured together using screws with a custom designed polydimethylsiloxane (PDMS) gasket (having a thickness of 1 mm) placed between them in order to ensure a tight seal and form a leak proof system. The gasket was designed such that it incorporated a raised lip (0.5 mm high) around each bioreactor array.

**Fabrication**

The two parts of the bioreactor array were fabricated by micromilling the required features into polycarbonate substrates. The gasket was fabricated by moulding of PDMS in a custom milled polycarbonate mould. Vial trays capable of housing 32 vials (16 for holding cell culture media and 16 for storage of the waste media), were fabricated in 5 mm sheets of polymethylmethacrylate (PMMA) using a CO_2_ laser cutter (Epilog Mini 18 Laser, CO 80403, USA).

**Viability determination of hPCLS**

ATP content of hPCLS was assessed according to the manufacturer’s protocol of the ATP Bioluminescence Assay Kit CLS II (Roche, Mannheim, Germany) in a black 96-well plate in the Lucyl luminometer (Anthos, Durham, NC) using a standard ATP calibration curve. The ATP content was normalized for protein content of the hPCLS as described below. Morphology was assessed on 4 μm sections of formaldehyde fixated, paraffin-embedded slices, stained with hematoxylin and eosin according to the described protocol (de Graaf *et al.*, 2010).

**Protein Content of hPCLS and iPSC-derived hepatocytes in the scaffold**

The pellet left from homogenized ATP samples was used to determine the protein content of hPCLS according to Lowry by using the Bio-Rad DC Protein Assay (Bio-Rad, Munich, Germany) as described before using bovine serum albumin for the standard curve. Protein content of cells in the scaffold was measured according to the manufacturer instructions of Pierce BCA Protein Assay Kit (cat. no. 23227) after protein extraction by an over-night incubation of the scaffold in 0.2 M NaOH. More information is found in Supplementary Figure S5.

**Gene expression analysis**

Total cellular RNA was purified by using the RNeasy Micro kit (Qiagen, 74004). Differentiated cells were lysed directly in the scaffold in the bioreactor using the lysis buffer provided in the Qiagen RNeasy Micro kit. The lysate was collected in microtubes and purified according to manufacturer’s instructions (Qiagen, 12/2007). Total RNA from the hPCLS was isolated using Maxwell 16 LEV simplyRNA Tissue Kit (Promega, USA). The RNA was converted to cDNA using the High Capacity cDNA Reverse Transcription Kit (Applied Biosystems, 4374966) according to the manufacturer’s instructions (06/2010). Quantitative real time PCR was conducted using the TaqMan Gene Expression Assays (Applied Biosystems 4331182), ALB (albumin) ID: Hs00910225_m1, AFP (alpha-fetoprotein) ID: Hs00173490_m1, CYP2B6 ID: Hs04183483_g1, CYP3A4 ID: Hs00604506_m1, CYP3A5 ID: Hs00241417_m1, CYP3A7 ID: Hs00426361_m1, HNF4A (hepatocyte nuclear factor-4-alpha) ID: Hs00230853_m1, NR1I3 (CAR, constitutive androstane receptor) ID: Hs00231959_m1, ABCB11 (BSEP, Bile Salt Export Pump) ID: Hs00184824_m1, ABCB1 (P-gp, permeability glycoprotein) ID: Hs00184500_m1, and KRT7 (cytokeratin-7) ID: Hs00559840_m1), TaqMan Gene Expression Master mix (Applied Biosystems, 4370048) and RNase-free water according to the manufacturer’s instructions (Applied Biosystems 11/2010). The respective C_t_ values obtained after analysis in a Chroma4 real time PCR machine (MJ Research, the program run at 50ºC for 2 minutes, 95 ºC for 10 minutes and 40 cycles of 15 sec at 95 ºC and 1 minute at 60 ºC) were normalized to the C_t_ value of CREBBP (CREB-binding protein) ID: Hs00231733_m1. CREBBP has been shown to be a good candidate to use for normalization since the gene expression does not change significantly during differentiation (Synnergren *et al.*, 2007). The gene expression is presented as individual data points using the formula: 2^-∆Ct^.

**Imaging**

Phase contrast images of 2D flow cultures were acquired by a Zeiss Axio Observer.Z1 microscope equipped with a 10x/0.3 Plan-Neofluar objective, and a Zeiss Axiocam MRm B/W camera. A scan of each cell culture chamber was recorded with an exposure time of 5 msec. All images were acquired with a z-stack of 5 image planes (6 µm between each image plane). The images were processed by applying the AxioVision Extended Focus module on the z-stacks to obtain the best focused image, stitching the individual images together and finally converting the stitched images to one image.

Fluorescence based imaging of a cross section of the scaffold was carried out at day 22 of the cell culture differentiation. Scaffolds were sectioned longitudinally (along the axis of flow). Each scaffold was stained with either Höechst for showing cell distribution or Calcein AM for live cell imaging. Non-fluorescent Calcein AM is converted to highly fluorescent Calcein by intracellular esterase activity and stains viable cells green. Cells were imaged using an inverted microscope (Zeiss Axio Observer) using the appropriate excitation lights and filters.

**Confocal microscopy**

Scaffolds were sectioned longitudinally (along the axis of flow) on day 22 of the cell culture differentiation. Each sample was fixed in 3% paraformaldehyde (in PBS) for 10 minutes and permeabilized with Triton X-100 for 5 minutes. Subsequently, they were stained for 30 minutes with either Höechst 33342 (Invitrogen) for labeling the cell nuclei as well as Phalloidin (F432, Invitrogen) for labeling the F-actin. Confocal acquisitions were performed using a Zeiss LSM 700 module in the Axio Imager M2 upright microscope using a 40x/1.20 W Korr C-Apo objective. The confocal settings were as follows, section thickness 0.8 µm, pixel dwell 0.79 µs, pixel size 145 nm, optimal Z section number determined by the confocal software. To eliminate any possible cross-talk between channels, images were collected with a sequential scan, using the following laser lines and mirror settings: 488(30%) 495-560nm; 555(30%) 605-700nm.

**Albumin synthesis**

Albumin production was measured using the Human Albumin ELISA kit (Bethyl Laboratories, Mongomery, USA) according to the supplier’s recommendations. In brief, medium was collected from the well plate (static conditions) after 24 of incubation or at the outlet tubes (perfused systems) for 24 hours from both differentiated cell and hPCLS cultures and stored at -20°C until analysis. Samples were diluted if necessary and human albumin was used to prepare a calibration curve. The amount of albumin was calculated based on a standard curve generated as a 4-parameter curve fit. Values are expressed as ng albumin produced per hour, per milligram total protein.


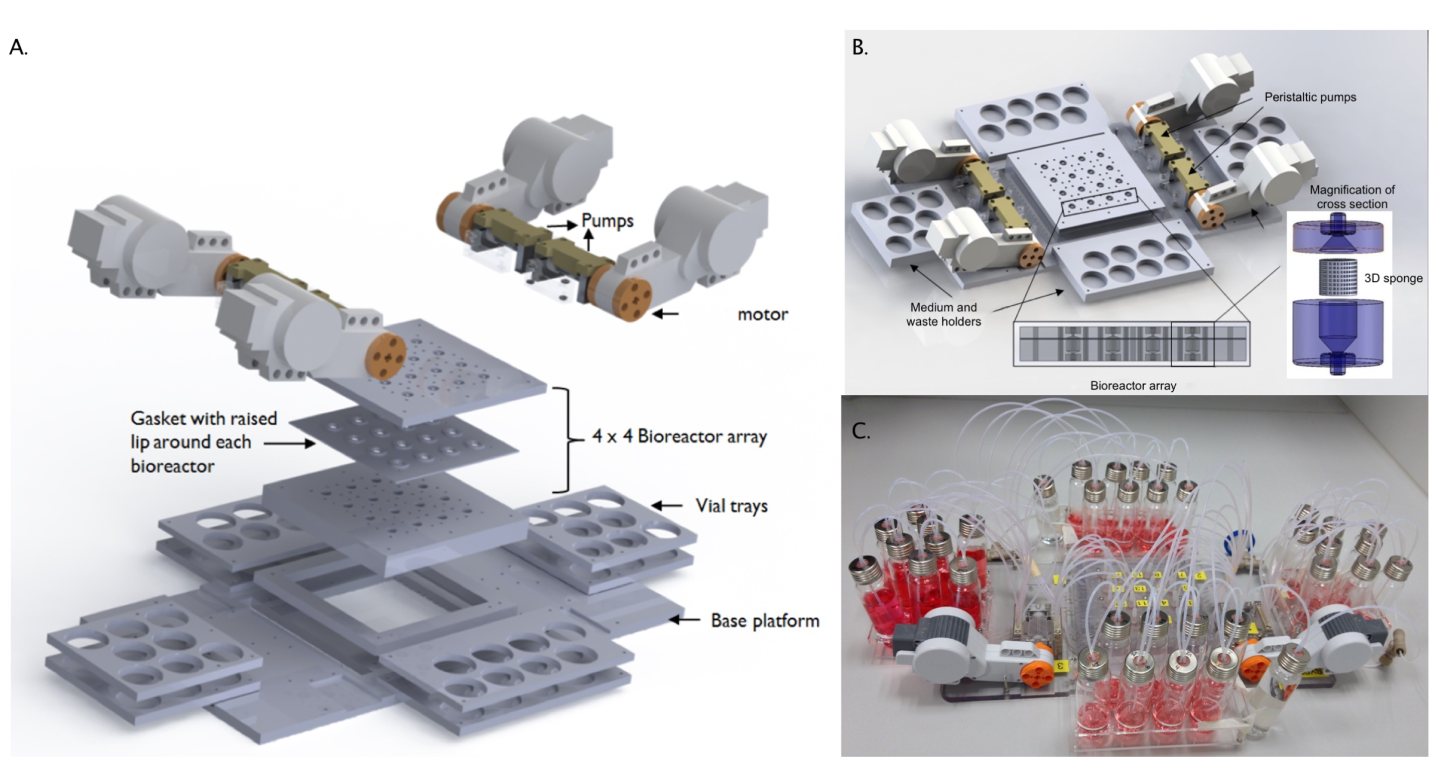


**Supplementary** **Figure S1. System description.** A: Parts of the 3D perfusion system. B: Render of assembled 3D perfusion system. C: Picture of a perfusion system with tubes and vials.


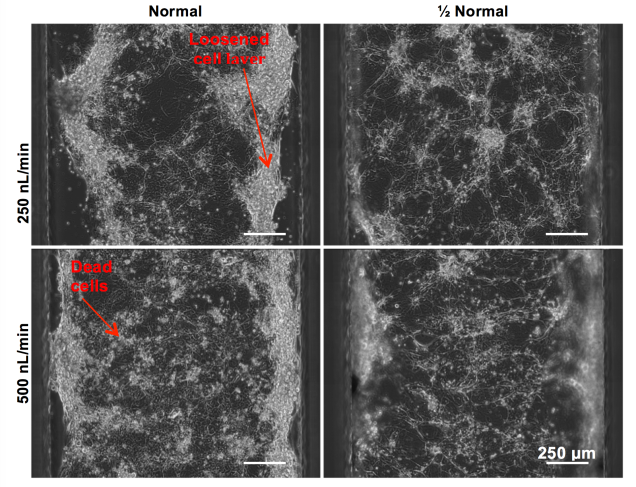

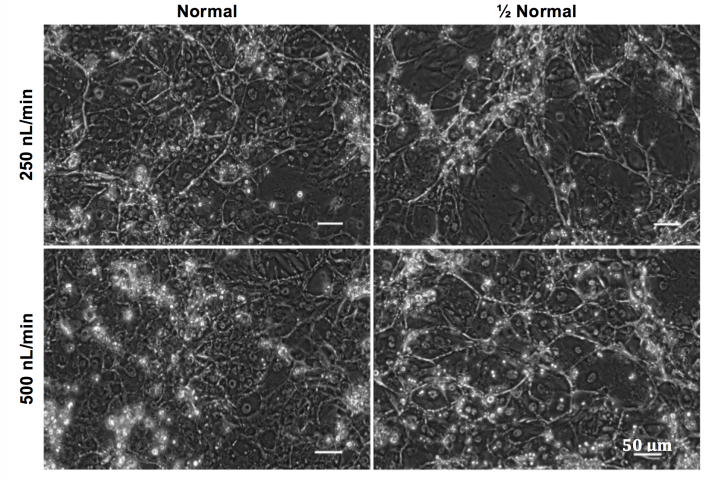


**Supplementary** **Figure S2. Effect of concentration of signaling factors on cell morphology at flow conditions.** Left panels: low magnifications, right panels high magnifications. DE cells were cultured and differentiated at 2D flow conditions in a chamber having dimensions of 1.5 mm (*w*) x 6 mm (*l*) x 0.5 mm (*h*). The concentration of signaling factors was either the normal used and optimized for static culture conditions or ½ the normal concentration but otherwise a full base medium. The cells were perfused at two different flow rates, 250 nL/min or 500 nL/min, corresponding to an exchange of the medium in the entire chamber every 20 and 10 minutes. Phase contrast images acquired at day 25. The shown images are a representative area of one chamber out of 4 chambers for each condition. A better cell attachment and less dead/floating cells were observed at perfusion with medium supplemented with half of the normal concentration of signaling factors compared to perfusion with medium supplemented with the normal concentration of signaling factors.

**
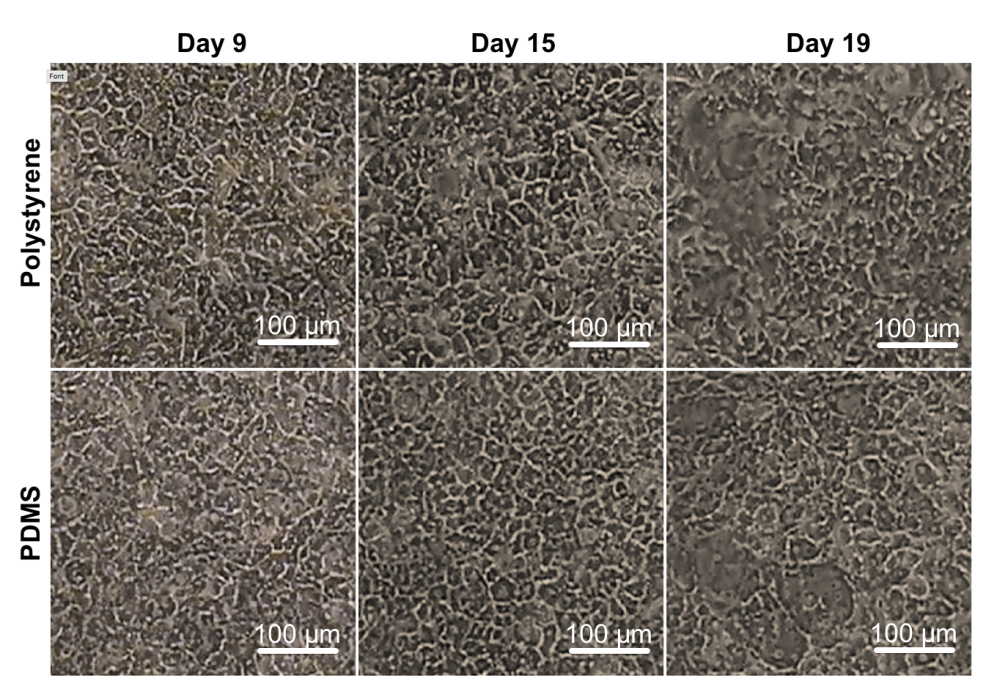
**

**Supplementary** **Figure S3. Cell morphology of differentiating cells at static conditions.** Phase contrast images were acquired at day 9, 15, and 19. The cell morphology was very similar between the PDMS scaffold material and the PS conventional surface substrate, although some larger cells were observed on PDMS)

**
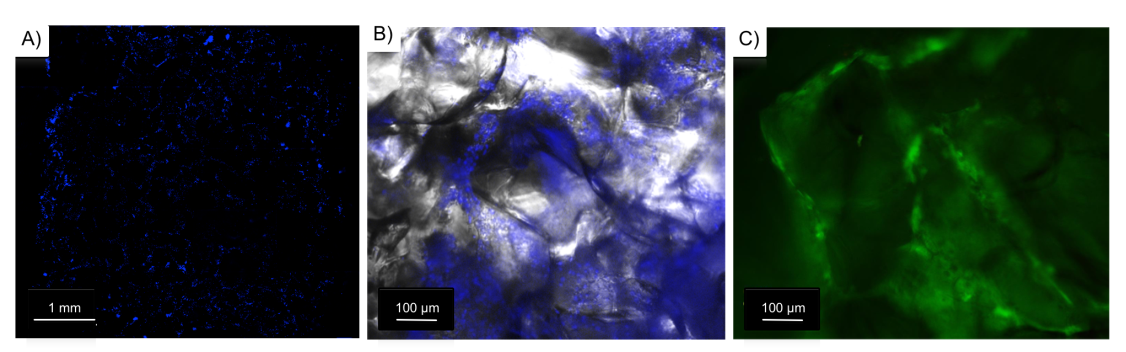
**

**Supplementary** **Figure S4.** Microscopy imaging of hiPSC-derived DE cells cultured and hepatic differentiated inside a porous scaffold at perfusion conditions: Image at day 22 after cell seeding of the middle of a cross-sectioned scaffold. A) Scan of entire cross-sectioned scaffold showing cell distribution. Höechst stained cell nuclei in blue color. B) Close-up view of cell distribution with Höechst stained cell nuclei. The homogenous blue fields in the image are likely not cells but reflections within the scaffold. The fluorescence image is merged with a phase contrast image of the scaffold. C) Calcein-AM live-stained cells in green.

**Supplementary** **Figure S5. Determination of protein content in the BAL.** DE cells were loaded into the BAL as described in material and methods. Three replicates of the four investigated BALs (two different scaffold designs and two different flow conditions), where prepared and cultured for one week. Scaffolds with cells were perfused with Dulbecco’s Phosphate Buffered Saline with MgCl_2_ and CaCl_2_ (Sigma D8662) for 30 min to remove protein containing medium and then transferred to an 1.5 mL Eppendorf tube with 0.5 mL 0.2M NaOH. To enhance distribution of NaOH within the scaffold, the tubes were vortexed for 30 seconds three times with 10 minutes incubation between each vortex. The tubes were incubated over night at 4°C. To enhance the release of cell material from the scaffold into the NaOH, the tube was again vortexed for 3 X 30 seconds with 10 minutes incubation between each vortex, and then centrifuged at 500 x g for 5 minutes. The supernatant was diluted 1:1 with milliQ water to 0.1M NaOH. The protein content was measured by the use of the Pierce BCA Protein Assay Kit (cat. no. 23227) according to the supplier’s microplate procedure. The absorbance was read at 570 nm and protein calculated based on a standard curve for bovine serum albumin. The results showed that the variance between the same type of scaffold was limited. If it is assumed that 0.1 mg protein corresponds to 100.000 cells (Anders Aspegren, personal observations), each BAL contains about 200.000-270.000 cells. This corresponds well with theoretical calculations that there can be a maximum of about 600,000-1,000,000 cells per BAL (see Supplementary Figure S6).


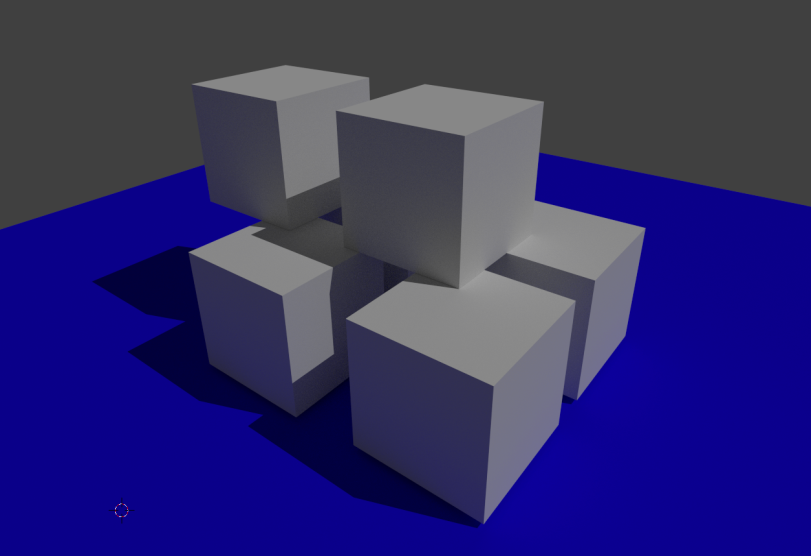


**Supplementary** **Figure S6.** Theoretical calculations of the surface area of a scaffold. Two estimates were made: **Estimate 1** was based on an idealized network of cubes that are connected and then surrounded by PDMS. The calculation involved trying to find out how many salt cubes that can fit into the volume of the scaffold and then calculate the surface area by taking the number of salt particles multiplied by the total surface of one salt particle. The side of a salt cube is approximately 0.35 mm as determined by scanning electron microscopy investigations. The distance to the next salt particle is estimated to be 0.1 mm (can also be larger) meaning that a salt crystal takes up about 0.45*0.45*0.45= 0.091125 mm^3^ including the surrounding PDMS. The cylinder volume (Figure 1C) is r^2^*pi*h=3*3*pi*5=141mm^3^. 141/0.091125= 1556 particles. The surface area of a salt particle is 0.35*0.35*6=0.735 mm^2^. 1556*0.735= 11.39 cm^2^. However because the salt crystals need to touch each other in order to form a network, some of the area is lost. In the idealized situation, each cube loses about 1.5 sides in surface area as it shares that area with other salt crystals. Therefore the area is estimated to be 11.39*9/12=8.5 cm^2^ In **Estimate 2** we used the measured porosity (determined to be 65%) of a random scaffold as input parameter and calculated the number of salt molecules that could fit into pores with a total volume of 0.65 multiplied by the scaffold volume: r^2^*pi*h*0.65=3*3*pi*5*0.65=92 mm^3^. Volume of salt particle is 0.35*0.35*0.35mm^3^=0.042875mm^3^. Number of salt particles in scaffold are 92/0.042875 = 2145 particles. Number of particles multiplied by the surface of each salt cube (6*0.35*0.35mm^2^=0.735mm^2^) = total surface volume: 1577 mm^2^=15.8 cm^2^. However, just as in the case above, some of the sides of the cubes are shared between each sugar cube. Using the estimate above, it is suggested that the surface area is 15.8 cm^2^*9/12= 11.85 cm^2^. In this case, the surface area is estimated to be about 12 cm^2^, which is close to **Estimate 1** of 11.39 cm^2^. For simplicity we estimate the surface of a scaffold to 10 cm^2^ (1000 mm^2^).

The number of cells fitting the scaffold is proportional to the surface of one side of the cuboidal cell. A 20 µm x 20 μm cell surface will result in a theoretical cell limit of 1000/(0.02*0.02)=2.5 million cells per scaffold. This is given that the cell surface area is the same in the 3D scaffold as on the 2D batch culture. Typically 80,000-100,000 iPSC-differentiated hepatocytes can be harvested per cm^2^ cell culture dish indicating that the maximal total cell number in the scaffold is between 800,000-1,000,000 cells.


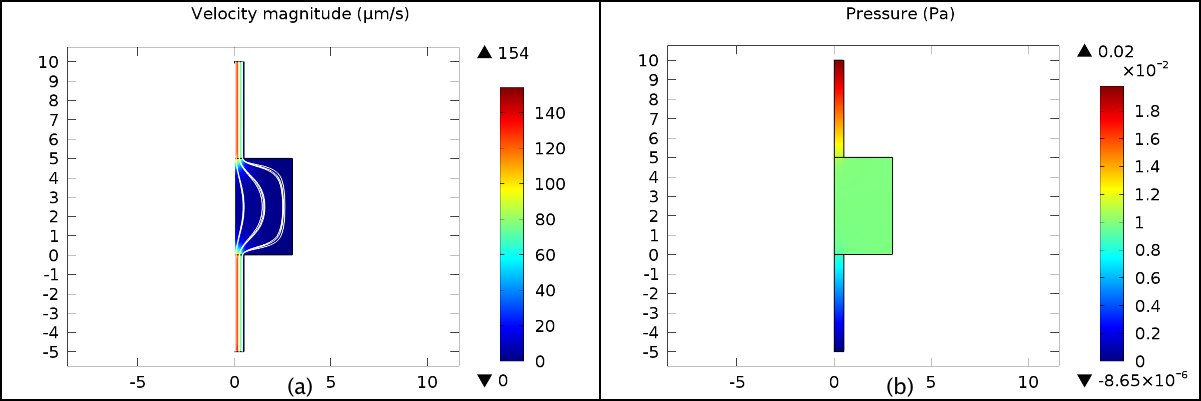


**Supplementary** **Figure S7.** Evaluation of shear stress acting on the 3D scaffold at the microscale.

Velocity profile (a) and pressure gradient (b) within the bioreactor as well as in the 3D scaffold. Different COMSOL simulations were performed to calculate the shear stress that cells sense within the 3D scaffold. The first analysis was made at the macro scale, evaluating the velocity field and pressure gradient in the reactor as well as in the 3D porous scaffold.

The domain region outside the scaffold was modeled by using the incompressible Navier-stokes equation, which describes how the velocity, pressure, temperature and density of a moving fluid are related and include the effects of viscosity on the flow.

$\rho\frac{\partial u}{\partial t}-\eta\nabla^{2}u+ \rho\left( u\cdot\nabla\right)u + \nabla p= F$ (1)

$\nabla\cdot u=0$ (2)

where, ρ is fluid density (kg$\cdot$m^-3^), u is the fluid velocity (m$\cdot$s^-1^), η is viscosity (Pa$\cdot$s), p is the pressure (Pa), F the volume force (N), and ∇ the standard del (nabla) operator.

The region containing the porous scaffold was modeled by using the Darcy-Brinkman equation, assuming that the scaffold has uniform microarchitecture and cylindrical pores.

$\mu\nabla^{2}u_{s}- \frac{\mu}{\kappa}u_{s}=\nabla$ (3)

$\nabla u_{s}=0$ (4)

where μ is the effective viscosity of the porous medium (Pa$\cdot$s), u_s_ is the fluid velocity (m$\cdot$s^-1^), p is the fluid pressure (Pa) and κ is the permeability of the porous scaffold (m^2^).

A flow rate of 3.3 µl/min was applied to the inlet, the boundary condition at the outlet was set at zero pressure, and the no slip boundary conditions were used along the walls of the model.

| 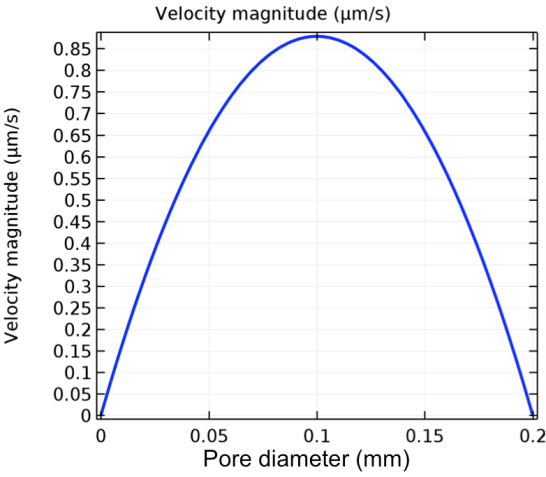  (a) | 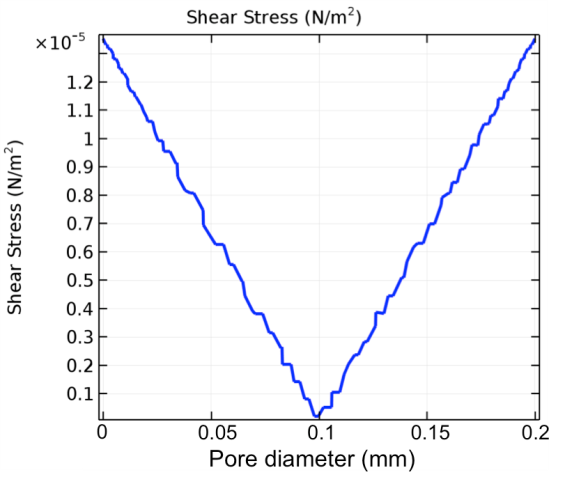  (b) |
| --- | --- |

**Supplementary** **Figure S8.**

Velocity profile (a) and shear stress (b) profile inside of a single channel having a pore diameter of 200μm within the 3D porous scaffold. The model shows that with a pore diameter of 200μm the shear stress acting on the wall is 1.2∙10^-5^N∙m^-2^, which is beyond the shear stress limit that leads hepatocytes to death. To evaluate the magnitude of the shear stress acting on the walls of the porous channels within the 3D scaffold, an analysis at the micro-scale was assessed.

If we considered the scaffold as a cylindrical structure with interconnected channels with different pores diameters, the flow rate for a single channel is described by the Fanning equation:

$Q_{i}=\frac{\pi D_{i}^{4}\Delta P}{128\mu L}$ (5)

where μ is the medium viscosity and L and D_i_ are the channel length (that is the scaffold height), and the channel diameter respectively and ∆P is the total pressure drop. The total pressure drop was evaluated in the analysis at the macro-scale, and the mean pore diameter was evaluated experimentally and it was found to be 200μm (Mohanty *et al.,* 2016) The single channel was modeled with the equation (1) and (2), by using as input the flow rate calculated with the equation (5). For a channel having the diameter of 200μm, the shear stress acting on the walls, which correspond to the domain where the cells formed focal adhesions, is approximately 1.2∙10^-5^N∙m^-2^.


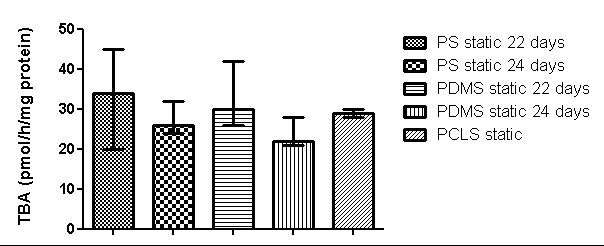


**Supplementary** **Figure S9. Bile acid production by hiPSC-derived hepatocytes and human PCLS.** Graph represents mean values ± SEM. Total bile acid (TBA) content was measured using the Total Bile Acid kit (Diazyme Laboratories, Poway, CA, USA) in medium after 24 h incubation (static cultures) or 24 h perfusion (perfused cells and slices). 1ml of medium was concentrated 10 times using the CentriVap Benchtop Vacuum Concentrator at 35°C (Labconco, Kansas City, MO, USA). TBA content was determined according to the manufacturer’s protocol of the TBA kit with a few modifications. Conjugated cholic acid (50µM) was used as a calibrator. Measurement was performed at 37°C in a 96-well plate in the Synergy HT plate reader (BioTek, Winooski, VT, USA). The absorbance was read at 405nm at 5 and 30 min. The TBA production is expressed as median with interquartile range.


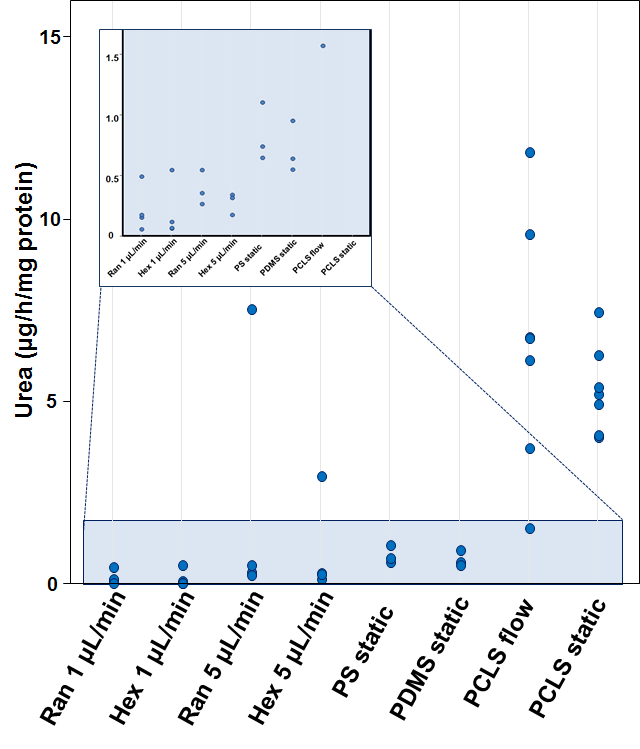


**Supplementary** **Figure S10. Urea production.**

Urea concentrations in the medium were measured using the Urea Assay Kit (Abnova, Taiwan). Medium samples from differentiated cells and PCLS flow and static cultures were concentrated 10 times before measurement using the CentriVap Benchtop Vacuum Concentrator at 35°C. Urea content was determined according to the manufacturer’s protocol of the Urea Assay kit with a few modifications. Accordingly, 25µL of samples were added to each well and incubated for 30 min at room temperature with a reagent mix. The absorbance was read at 430nm and urea levels calculated based on standard curve of urea standard provided with the kit and expressed as μg urea produced per h, per mg total protein. Data are expressed as individual values.

**REFERENCES**

de Graaf, I. A. M., Olinga, P., de Jager, M. H., Merema, M. T., de Kanter, R., van de Kerkhof, E. G., & Groothuis, G. M. M. (2010). Preparation and incubation of precision‐cut liver and intestinal slices for application in drug metabolism and toxicity studies. Nat Protocols, 5, 1540–1551.

<https://doi.org/10.1038/nprot.2010.111>

Mohanty, S., Sanger, K., Heiskanen, A., Trifol, J., Szabo, P., Dufva, M., … Wolff, A. (2016). Fabrication of scalable tissue engineering scaffolds with dual‐pore microarchitecture by combining 3D printing and particle leaching. Materials Science and Engineering: C, 61, 180–189.

https://doi.org/10.1016/j.msec.2015.12.032

Patrachari, A. R., Podichetty, J. T., & Madihally, S. V. (2012). Application of computational fluid dynamics in tissue engineering. *Journal of Bioscience and Bioengineering*, *114*, 123–132.

<https://doi.org/10.1016/j.jbiosc.2012.03.010>

Skafte‐Pedersen, P., Sabourin, D., Dufva, M., & Snakenborg, D. (2009). Multi‐channel peristaltic pump for microfluidic applications featuring monolithic PDMS inlay. *Lab on a Chip*, *9*, 3003–3006.

<https://doi.org/10.1039/b906156h>

Synnergren, J., Giesler, T. L., Adak, S., Tandon, R., Noaksson, K., & Lindahl, A. (2007). Differentiating human embryonic stem cells express a unique housekeeping gene signature. *Stem Cells*, *25*, 473–480.
